# 1827. Patients with Serious Injection Related Infections (SIRI) have Limited Access to Community-based Infection Prevention and Treatment in Alabama

**DOI:** 10.1093/ofid/ofad500.1656

**Published:** 2023-11-27

**Authors:** William Bradford, Kelly Gagnon, John R Bassler, Ellen Eaton

**Affiliations:** University of Alabama Birmingham, Birmingham, Alabama; University of Alabama Birmingham, Birmingham, Alabama; University of Alabama at Birmingham, Birmingham, Alabama; University of Alabama, Birmingham, Birmingham, Alabama

## Abstract

**Background:**

The University of Alabama at Birmingham Hospital (UABH) provides a large percentage of tertiary care for Alabamians, including treatment of serious injection-related infections (SIRI). Because patients with SIRI frequently utilize our hospital and emergency department, we hypothesized that persons who inject drugs (PWID) in AL have limited access to community-based infection prevention and treatment, including addiction and harm reduction services, that is associated with their rural residence.

**Figure:**
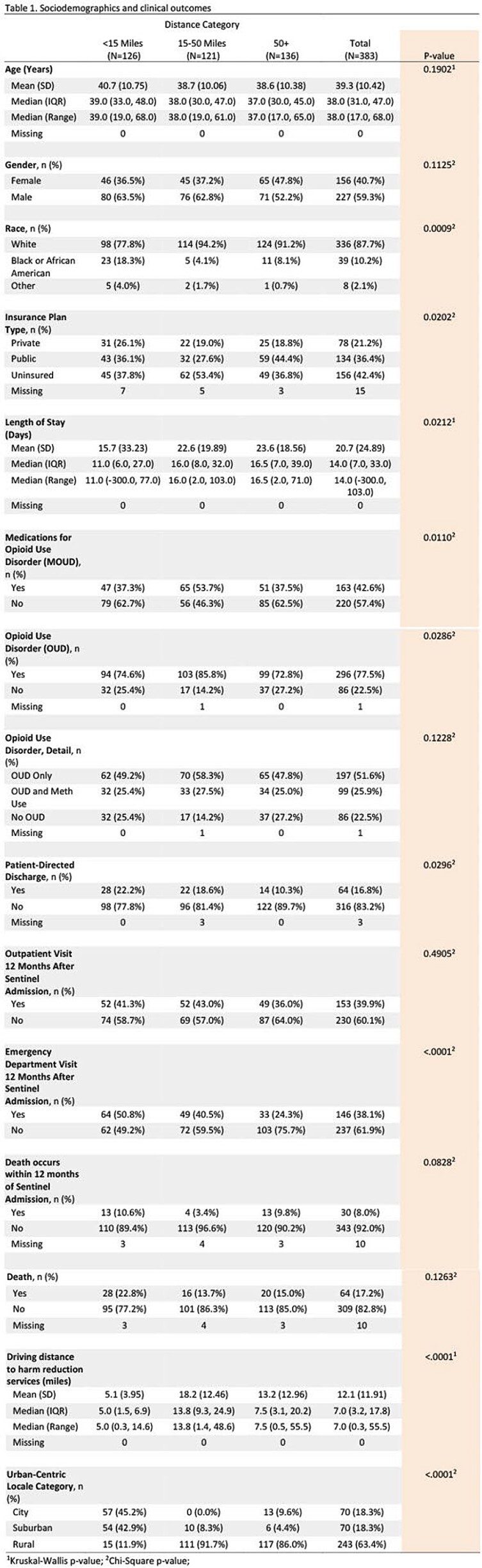

Sociodemographics, healthcare utilization, infection type, and substance use outcomes of participants.

**Methods:**

We conducted a retrospective cohort study of patients hospitalized with SIRI at University of Alabama at Birmingham (UAB) Hospital (2016 to 2021). We queried sociodemographics, healthcare utilization, infection, and substance use outcomes (table 1). We used a standardized telephone survey, to identify and characterize the community services for PWID in AL. We then categorized the distance between self-reported residence, using zip code, and two locations: UAB hospital and community services. Using geospatial mapping, we then evaluated patients’ driving distances to these sites.

**Results:**

A total of 383 patients with SIRI met inclusion criteria (table 1). We identified 98 sites providing community services (HIV, harm reduction, addiction) for PWID in AL. Patients residing in a community at intermediate distance from UAB (defined as 15-50 miles) had the most limited access to services. They were also significantly more likely to be white, uninsured, rural, have an established diagnosis of opioid use disorder (OUD).

**Conclusion:**

For patients with SIRI receiving care at UAB Hospital, the proximity of their residence to UAB was associated with access to community services, with those 15 to 50 miles away being most limited. Although this group has access to care when hospitalized, the results highlight the lack of community care available in their hometowns. Clinicians should anticipate this gap in care and arrange for telehealth and other accessible options; integrated infection and addiction telehealth services may help bridge this care gap. More research is needed to understand how the residence of PWID may impact post-hospital ID and addiction outcomes.

**Disclosures:**

**Ellen Eaton, MD, MPH**, Gilead: Grant/Research Support|Gilead: Honoraria

